# Azole Resistance in *Aspergillus fumigatus*: A Five-Year Follow Up Experience in a Tertiary Hospital With a Special Focus on Cystic Fibrosis

**DOI:** 10.3389/fcimb.2020.613774

**Published:** 2021-02-18

**Authors:** Hélène Guegan, Emilie Prat, Florence Robert-Gangneux, Jean-Pierre Gangneux

**Affiliations:** ^1^ Laboratoire de Parasitologie-Mycologie, Centre Hospitalier Universitaire de Rennes, Rennes, France; ^2^ Univ Rennes, CHU, Inserm, Irset (Institut de Recherche en santé, environnement et travail) – UMR_S 1085, Rennes, France

**Keywords:** azole resistance, *cyp51A*, *Aspergillus fumigatus*, cystic fibrosis, invasive aspergillosis, hematology, intensive care unit

## Abstract

Azole-resistant *Aspergillus fumigatus* (AR*Af*) has emerged worldwide during the last decades. Drug pressure after long term treatments of chronically infected patients and the propagation of environmental clones selected under the pressure of imidazoles fungicides used in agriculture and farming both account for this emergence. The objectives of this study were to determine the rate of azole resistance in *Aspergillus fumigatus* during a 5-year period, taking into account (i) differences between underlying diseases of the patients treated, (ii) cross-resistance between azoles, and (iii) focusing on the 5-year evolution of our center’s cystic fibrosis cohort. Overall, the rates of voriconazole (VRC)-resistant and itraconazole (ITC)-resistant *A. fumigatus* isolates were 4.1% (38/927) and 14.5% (95/656), respectively, corresponding to 21/426 (4.9%) and 44/308 (14.3%) patients, respectively. Regarding cross-resistance, among VRC-R isolates tested for ITC, nearly all were R (20/21;95%), compared to only 27% (20/74) of VRC-R among ITC-R isolates. The level of azole resistance remained somewhat stable over years but greatly varied according to the azole drug, patient origin, and clinical setting. Whereas azole resistance during invasive aspergillosis was very scarce, patients with cystic fibrosis were infected with multiple strains and presented the highest rate of resistance: 5% (27/539) isolates were VRC-R and 17.9% (78/436) were ITC-R. These results underline that the interpretation of the azole resistance level in *Aspergilllus fumigatus* in a routine setting may consider the huge variability depending on the azole drug, the clinical setting, the patient background and the type of infection.

## Introduction

*Aspergillus fumigatus*, a ubiquitously distributed opportunistic pathogen, is the leading agent of aspergillosis, ranking first among fungal killers. In profoundly immunocompromised patients, the clinical picture rapidly evolves towards an acute angio-invasive form or invasive aspergillosis (IA) that accounts for one of the major severe invasive fungal diseases (Ullmann et al., 2018). In the immunocompetent individual, the infection is generally limited to cavitating, chronically evolving forms (such as aspergilloma), chronic fibrotic or immuno-allergic forms (allergic bronchopulmonary aspergillosis and severe asthma with *Aspergillus* sensitization) (Denning et al., 2016). During cystic fibrosis, abnormally viscous bronchial secretions prevent mucociliary clearance and promote the trapping and the proliferation of inhaled bacteria and fungal spores (Prigitano et al., 2017). *A. fumigatus* is the most frequent filamentous fungus colonizing the airways of patients with cystic fibrosis (CF) followed by *Scedosporium* sp. (Pihet et al., 2009; Felton and Simmonds, 2014; Hamprecht et al., 2018). Recent publications underlined that patients with *Aspergillus* in the airway have greater abnormalities on CT imaging, particularly in children, at the time of infection and in the following years, as shown in longitudinal studies (Breuer et al., 2019).

Voriconazole (VRC), isavuconazole (ISA), itraconazole (ITC), and posaconazole (POS) are four triazole antifungals recommended as first-line drugs in the treatment or prophylaxis of aspergillosis. They inhibit the lanosterol 14-α-demethylase enzyme (Cyp51A) encoded by the *cyp51A* gene, thereby inhibiting the ergosterol synthesis. Voriconazole and isavuconazole are highly recommended for the treatment of IA (Ullmann et al., 2018). Posaconazole shows a very wide spectrum of activity and its primary clinical indications are i) salvage therapy for patients with IA and ii) prophylaxis in patients with neutropenia and hematopoietic cell transplantation (Ullmann et al., 2018). It has also been reported as an alternative treatment for ABPA in CF patients (Periselneris et al., 2019). Itraconazole is the oldest but still robust triazole drug used in chronic and immuno-allergic aspergillosis and allows minimizing the use of corticosteroids (Denning et al., 2016). Azole resistance in *A. fumigatus* isolates is increasingly reported with variable prevalence in the six continents (Chowdhary et al., 2013; Meis et al., 2016; Wiederhold and Verweij, 2020). Two main origins of the emergence of azole resistant *A. fumigatus* (*ARAf*) are recognized: (i) long-term use of triazoles in patients with chronic *Aspergillus* infections and (ii) increased use of agricultural fungicides against plant-pathogenic moulds, such as *Fusarium, Mycosphaerella* and *A. flavus*, with cross-activity against *A. fumigatus* (Meis et al., 2016).

A global rate of resistance of *A. fumigatus* is however a metric with little significance, especially if we do not take into account variability between patients and drugs. During CF, particularly-high rates of AR*Af* have been reported (Stevens et al., 2003; Mortensen et al., 2011; Burgel et al., 2012; Morio et al., 2012; Meis et al., 2016; Prigitano et al., 2017; Risum et al., 2020; Wiederhold and Verweij, 2020) while azoles are recommended in the guidelines for the management of allergic bronchopulmonary aspergillosis (ABPA) (Stevens et al., 2003).

The aim of this prospective study was to estimate the frequency of azole resistance in *A. fumigatus* isolated from patients in our tertiary care University hospital, over a 5-year period. In particular, we analyzed the prevalence of AR*Af* according to patient background and azole drug, the cross resistance between azoles, and the characteristics of the longitudinal carriage of *Aspergillus* in CF patients, and more particularly of AR*Af*.

## Methods

### Patients

Data of antifungal susceptibility testing performed on *A. fumigatus* clinical isolates as a part of the routine lab procedure between January 2015 and December 2019 were retrospectively collected. A total of 929 A*. fumigatus* isolates from 426 patients were tested for susceptibility to at least one azole. Among them, 595 isolates were obtained from sputum samples or throat swabs from 123 CF patients included in the cohorts of adult (N = 87) and pediatric (N = 36) “Centres de Ressources et de Compétences de la Mucoviscidose” at Rennes University Hospital (France). The remaining strains were isolated from patients hospitalized in ICU (N = 57), Pulmonology (N = 159), Hematology (N = 13) or other clinical wards (N = 105), and used for comparison to isolates from CF patients.

### Mycological Examination

(i) Culture and identification. All isolates of *Aspergillus fumigatus* were obtained after a culture of respiratory samples, using two Sabouraud dextrose agar slants supplemented with 0.5% chloramphenicol incubated at 30°C and 37°C for up to 7 days. Depending on the date of isolation, identification was performed using macroscopic and microscopic examinations of cultures, combined to MALDI-TOF mass spectrometry (MALDI Biotyper, Brucker France, Marne-la-Vallée) or molecular sequencing since 2018.
(ii) *In vitro* susceptibility to azoles. Itraconazole (ITC), voriconazole (VRC) and posaconazole (POS) minimum inhibitory concentrations (MICs) were determined using Etest^®^ strips and RPMI medium (Biomérieux, Marcy-L’Etoile, France) according to the manufacturer recommendations. MICs were determined following a 48 h incubation at 37°C. Strains with MIC ≥2 mg/L for ITC and VRC, and ≥0.25 mg/L for POS, were considered resistant (R), according to recent EUCAST breakpoints for fungi (European Committee on Antimicrobial Susceptibility Testing (EUCAST), 2020). Of note, isolates with MICs at 2 mg/L for ITC and VRC and 0.25 mg/L for POS (graded in ATU group, Area of Technical Uncertainty) remain suitable drugs for treatment depending on the clinical setting, following latest EUCAST recommendations (European Committee on Antimicrobial Susceptibility Testing (EUCAST), 2020). All other MICs classified isolates as susceptible (S), and therapeutic advice was given with the recommendation to use the tested drug.
(iii) Molecular characterization of *cyp51A* gene. While not performed routinely, the promoter and the whole *cyp51A* gene were sequenced in both strands from *A. fumigatus* isolates in some patients with repeated high MICs and/or clinical failure, using five sets of primers: PA5 and PA7 (Mellado et al., 2001), AF306F and AF855R, AF766F and AF1330R, AF1179F and AF1709R, and AF1426F and AF2025R (Alanio et al., 2011).

The PCR mixture contained 5 μl of DNA extract and 20 μl of a mix composed of 0.625 U of GoTaq^®^ Hot Start Polymerase (Promega, Charbonnières-les-Bains), 1x Colorless GoTaq^®^Flexi Buffer (Promega), 2mM of MgCl2 (Promega), 0.8mM of dNTP mix (Eurobio, Les Ulis), and 0.2 μM of each primer. The amplification program consisted of 5 min at 94°C, 30 cycles of 30 s at 94°C, 30 s at 58°C, and 1 min at 72°C, followed by a final step of 10 min at 72°C. Sequences of resistant strains were compared to the wild-type *A. fumigatus* sequence CM 237 (GenBank accession number AF338659), at http://blast.ncbi.nlm.nih.gov/Blast.cgi.

### Ethics and Statistics

According to the French Public Health Laws (Code de la Santé Publique, 2017), protocols of this type do not require approval from an ethics committee and are exempt from the requirement of formal informed consent. MIC values were expressed as median and interquartile range. Data analysis was performed using the GraphPad PRISM^®^ v.5.02 software. For comparison of proportion, the Fisher’s test was used. A p-value of 0.05 was considered statistically significant.

## Results

### Global Prevalence of Resistance in Azoles

Before 2015, *A. fumigatus* isolated in our center were considered as *a priori* susceptible to azoles and resistance monitoring with MICs was only performed in case of treatment failure. The growing number of treatment failures stressed the need to perform a systematic MIC determination to VRC and/or ITC, for each *A. fumigatus* isolated from 01/2015 to 01/2020. A total of 927 and 656 strains were evaluated for VRC and ITC susceptibility, respectively. Susceptibility to POS was tested in case of MIC ≥2 mg/L for VRC and ITC, or when the patient had a history of AR*Af* carriage.

**Figure 1:**
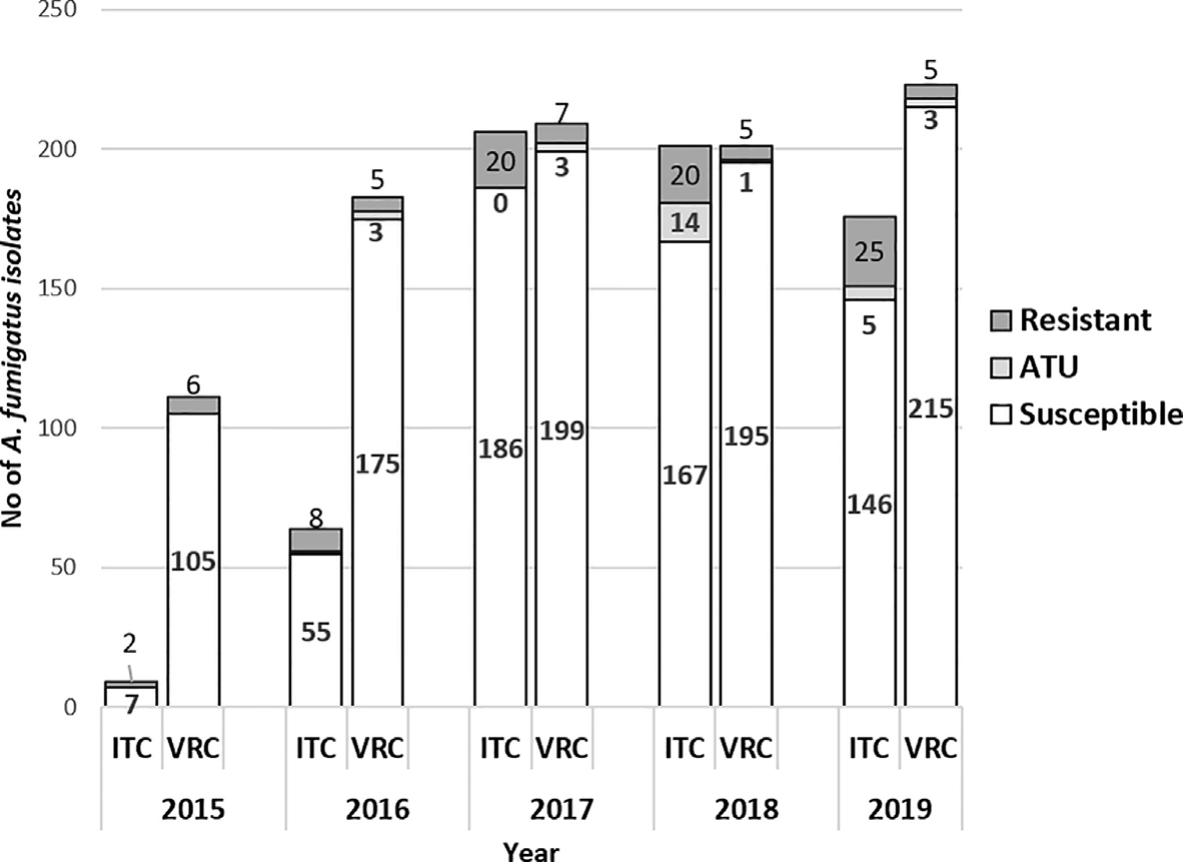
Number of *A. fumigatus* strains susceptible, ATU and resistant to ITC and VRC per year (2015–2019, N = 929) (VRC, n = 927); (ITC, n = 656). White bars indicate susceptible isolates; dark grey bars indicate resistant isolates with MIC >2mg/L and light grey bars represent isolates resistant isolates in ATU group (MIC = 2 mg/L). ITC, itraconazole; VRC, voriconazole; ATU, Area of Technical Uncertainty.

From 2015 to 2019, the rates of VRC-resistant and ITC-resistant *A. fumigatus* isolates were 4.1% (38/927) and 14.5% (95/656), respectively; they were recovered from 21/426 (4.9%) and 44/308 (14.3%) patients, respectively. During the last 4 years with an exhaustive dataset of VRC MIC determination (2016–2019), the rate of VRC-resistant *A. fumigatus* remained stable, with 3.8% (8/183), 4.8% (10/209), 3.0% (6/201), and 3.6% (8/223) (ns), with an overall median MIC at 4 [2; 8] mg/L (**Figure 1**). An exhaustive dataset was obtained during the last 3 years for ITC and showed a consistent rate at 9.7% (20/206) in 2017, increasing to 16.8% (34/201), and 17% (30/176) in 2018 and 2019, respectively (ns). The median MIC of ITC-resistant isolates was 14 [3; 32] mg/L. Among these resistant strains, 20/95 (21%) isolates and 10/38 (26.3%) had ITC MICs and VRC MICs in the ATU range, respectively, corresponding to 20/656 (3.0%) and 10/927 (1.1%) of all tested isolates for ITC and VRC, respectively (**Figure 1**).

### 2. Prevalence of Azole Resistance According to the Clinical Setting

#### Patients From the Cystic Fibrosis Cohort

**Figure 2:**
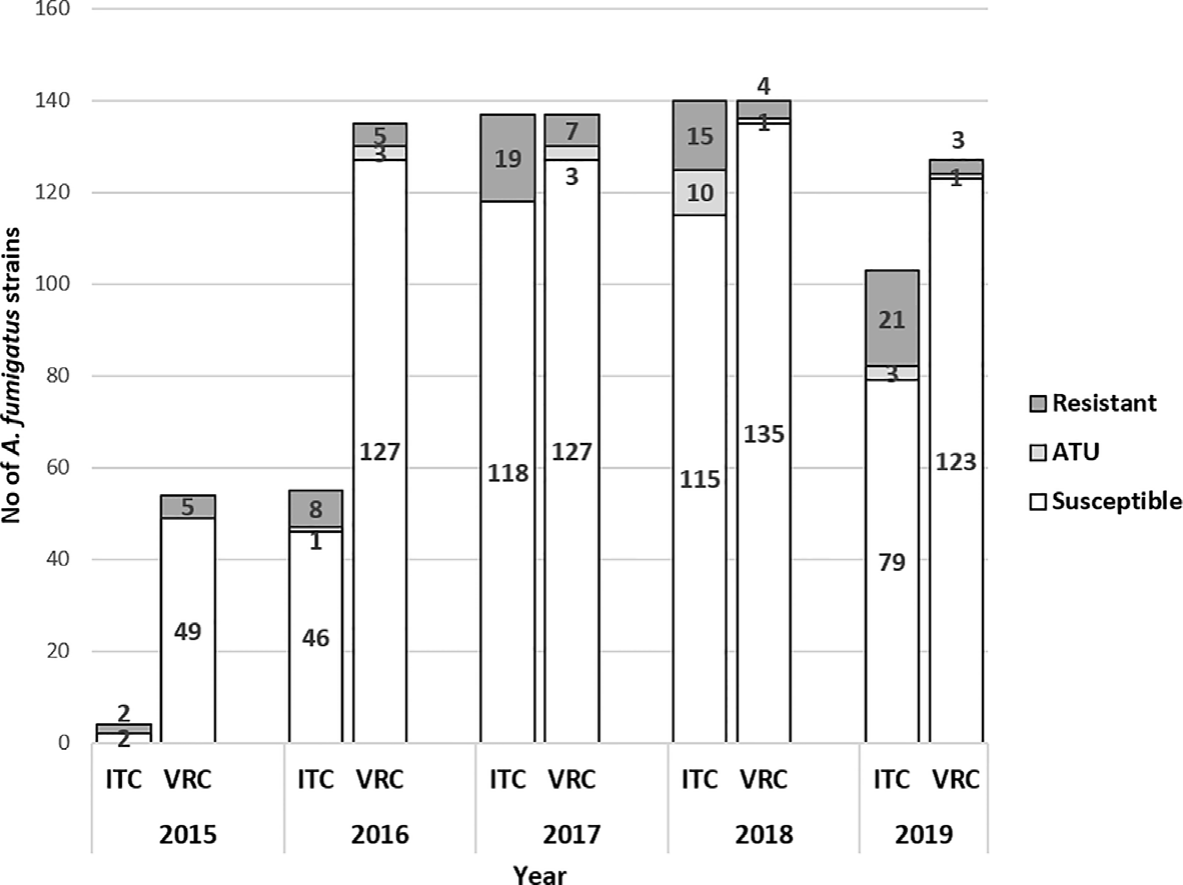
Number of *A. fumigatus* strains susceptible, ATU and resistant to itraconazole (ITC) and voriconazole (VRC) per year in CF patients (2015–2019, N = 595 (VRC, n = 593); (ITC, n = 440). White bars indicate susceptible isolates; dark grey bars indicate resistant isolates with MIC >2mg/L and light grey bars represent isolates resistant isolates in ATU group (MIC = 2 mg/L). ITC, itraconazole; VRC, voriconazole; ATU, Area of Technical Uncertainty.

CF patients are managed in special units. They are sometimes hospitalized in other Pulmonology units, but we have reclassified them as “CF patients” because this cohort of patients is followed individually. A total of 595 A*. fumigatus* isolates were collected from respiratory samples of 123 CF patients and screened for VRC and/or ITC and/or POS *in vitro* susceptibility, of whom 34 patients presented with at least one isolate resistant to one drug. The rate of isolates resistant to at least one azole reached 90/595 (15.1%) over the period 2015–2019, among which three isolates were only POS-R. Regarding VRC, the global frequency of resistant *A. fumigatus* strains from 2016 to 2019 was 5% (27/539), recovered from 13 out of 119 patients (10.9%). The rate of VRC-R isolates was relatively steady from 2016 to 2019 (**Figure 2**). Over the same period (2016–2019), the rate of ITC-R isolates was higher than that of VRC-R, with 78/436 (17.9%) resistant isolates, recovered from 29/111 (26.1%) patients. A tendency to increase was noticed, with rates of AR*Af* detection from 14 to 24% from 2017 to 2019, respectively.

#### Pulmonology

A total of 159 isolates from 143 patients hospitalized in pulmonology, excluding CF patients, were screened for azole susceptibility between 2015 and 2019. Only four isolates from four patients were resistant to ITC (including one ATU isolate), of which one was also resistant to VRC (**Table 1**). Another isolate was ATU to VRC only.

**Table 1:** Itraconazole (ITC) and voriconazole (VRC) resistance of *A. fumigatus* isolates from patients from pulmonology (excluding CF), intensive care unit, and other units.

Year	Number of *A. fumigatus* isolates resistant to antifungals* (n/N)					
	Pulmonology		ICU		Other	
	ITC	VRC	ITC	VRC	ITC	VRC
2015	0/1	0/30	nd	0/9	0/4	1/18
2016	0/2	0/21	0/2	0/6	0/5	0/21
2017	0/35	0/35	1/9	0/10	0/25	0/27
2018	4/31	1/31	4/15	0/15	1/15	0/15
2019	0/35	1/42	1/11	2/17	4/26	1/37
Total	4^1^/104	2^2^/159	6^3^/37	2/57	5^4^/75	2^2^/118^$^
%	3.8%	1.3%	16.2%	3.5%	6.6%	1.7%

ICU, intensive care unit; ITC, itraconazole; VRC, voriconazole; nd, not determined

*MIC ≥ 2 mg/L.

^$^Among them, 13 isolates were from hematology patients; 0/13 were resistant.

^1^Among them, 1/4 was ATU.

^2^Among them, 1/2 was ATU.

^3^Among them, 4/6 were ATU.

^4^Among them, 1/5 was ATU.

#### Intensive Care Units

Fifty-seven isolates from 50 patients were screened for azole resistance from 2015 to 2019. Six patients harbored an ITC-R isolate (of which 4 were ATU) and two isolates from one patient were VRC-R, none of them with azole cross resistance (**Table 1**). None of these patients were CF. A few patients with hematological malignancies have been occasionally hospitalized in ICU. We keep them as “ICU patients” as the specific patient management in ICU (mechanical ventilation for example) is different and can contribute to modify the epidemiology.

#### Other Wards

Cultural analysis in Hematology patients remains rare for the diagnosis of IA compared to galactomannan determination in the serum used for screening in high-risk patients. Only 13 A*. fumigatus* were isolated and tested in patients with hematological malignancies since 2015 and no azole resistance was detected. These patients are grouped with patients from other units in **Table 1**. In patients with miscellaneous clinical backgrounds (other units), five were infected with an ITC-R strain (co-resistant to VRC in one case), of which one was an ATU isolate.

### Cross-Resistance Between Azoles

We then analyzed whether there was cross-resistance between the main azole antifungals used routinely, in order to help for the decision of doctors excluding ATU isolates. Among the 83 A. *fumigatus* strains with high MIC for ITC and/or VRC (> 2 mg/L), 20 (24%) were resistant to both ITC and VRC. However, the proportions of ITC-R/VRC-S and ITC-S/VRC-R differed greatly. Among VRC-R isolates tested for ITC, nearly all were R to ITC (20/21;95%), while only 27% (20/74) of ITC-R isolates were VRC-R.

In our routine setting, POS is only tested in case of azole-resistance history or clinical failure after VRC or ITC treatment. Of the 56 isolates tested simultaneously for ITC, VRC and POS, 20 (36%) were susceptible to all of them (**Table 2**). A cross-resistance was observed in 54% (30/56) (**Table 2**). Two isolates had a decreased susceptibility to ITC only (MIC at 2 and 3 mg/L, respectively), and one isolate had an increased MIC for VRC only (at 2 mg/L). Among the 31 ITC-R isolates, 28 (90.3%) were POS-R whereas only 15 isolates (48.4%) were VRC-R. Overall, 76 isolates benefited from MIC evaluation to POS, of which 37 (48.7%) were POS-R. Thirty-four of the 37 (91.9%) isolates POS-R were also resistant to ITC and/or VRC, of which 32 were observed in CF patients.

**Table 2:** Prevalence of azole resistance in *A. fumigatus* isolates tested simultaneously for the three azole antifungals (N = 56).

Susceptibility profile	Azole antifungal			Number
	ITC	VRC	POS	
No azole R	S	S	S	20
R to 1 azole	**R**	S	S	2
	S	**R**	S	1
	S	S	**R**	3
R to 2 azoles	**R**	S	**R**	14
	S	**R**	**R**	1
	**R**	**R**	S	1
R to 3 azoles	**R**	**R**	**R**	14

R, resistance; S, susceptibility.

### Phenotypic and Genotypic Characterization of Isolates from CF Patients

**Figure 3:**
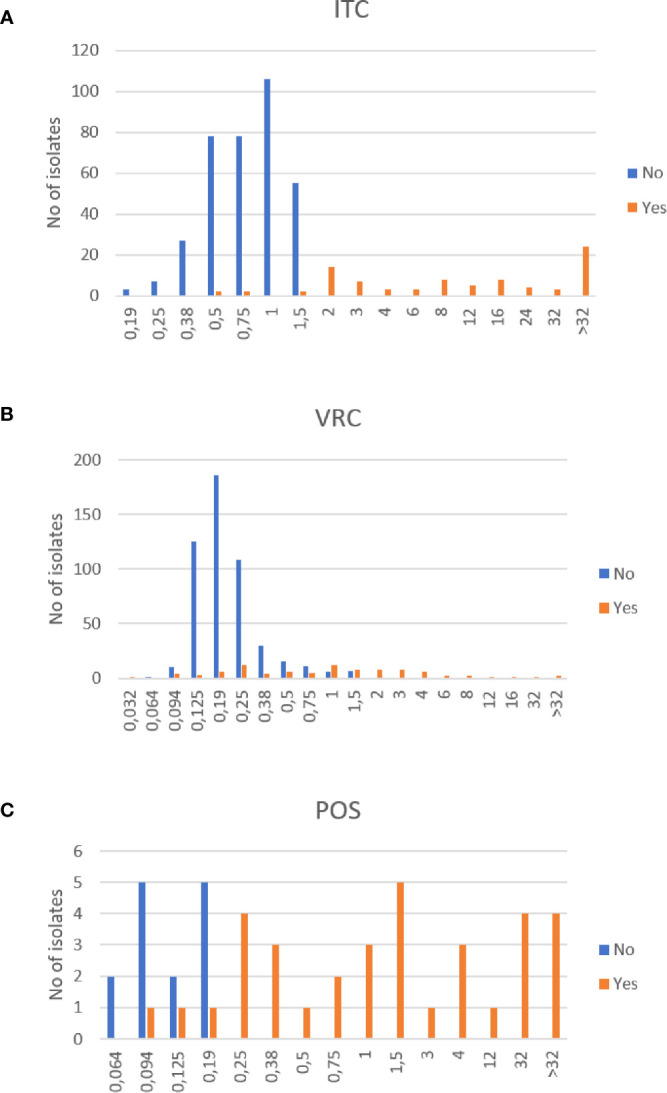
Distribution of azole MICs of *Aspergillus fumigatus* isolates from CF patients. Itraconazole (ITC) MICs **(A)**, voriconazole (VRC) MICs **(B)** and posaconazole (POS) MICs **(C)** of isolates with at least one resistance to one azole (Yes) or with no resistance to any azole (No). Unit: mg/L.

During the five-year follow-up, 34 out of 123 patients (27.6%) (90/595 isolates, 15.1%) presented at least once with an AR*Af* in induced sputum samples. About half of them (18/34) harbored a single AR*Af* among several *Aspergillus* isolates (2 to 18 consecutive isolates), of which seven were ATU (**Table 3**), whereas three patients had a single positive sputum with AR*Af* and no further positive *Aspergillus* cultures (patients #1, #13, and #25, **Table 3**). As seen in **Table 3**, the range of ITC and VRC MIC for different AR*Af* from a same patient was sometimes huge, suggesting that some patients are colonized with a multiplicity of strains over time. The MIC distribution of all isolates collected from patients with at least one AR*Af* is shown in **Figure 3**.

**Table 3:** Characteristics of isolates obtained from CF patients with at least one resistance to VRC or ITC over the study period (2015–2019).

No	No ofisolates	No of ITC-Risolates	MIC range of ITC-R isolates	No of VRC-R isolates	MIC range of VRC-R isolates	Mean time between isolates (days)	Preexposure to azole
1	1	ND	ND	1	4	NA	ND
2	18	11*	2;>32	10**^#^	2;>32	84	yes
3	16	4*	2;>32	1	16	101	yes
4	3	1	2	0	NA	458	yes
5	6	4	4;16	0	NA	74	yes
6	4	1	4	0	NA	86	no
7	14	1	2	1^#^	2	73	no
8	13	1	3	0	NA	104	yes
9	3	1	>32	1	> 32	89	yes
10	9	1	2	0	NA	185	no
11	3	2	>32	1	3	77	yes
12	11	1	2	0	NA	135	no
13	1	1	>32	0	NA	NA	no
14	6	1	24	0	NA	115	ND
15	7	3*	2;12	0	NA	160	yes
16	2	1	16	0	NA	730	ND
17	10	3	3;>32	3*	2;>32	146	yes
18	2	1	8	0	NA	1267	yes
19	3	1	8	0	NA	109	no
20	3	1	16	2	3;12	119	no
21	13	1	2	0	AN	125	ND
22	5	1	8	0	NA	195	yes
23	5	5	8;32	1	4	210	yes
24	14	9	2;>32	0	NA	114	yes
25	1	1	16	1	3	128	yes
26	9	5	6;>32	2*	2;8	169	yes
27	4	2*	2;3	0	NA	54	no
28	8	0		1	2	169	no
29	3	1	2	0	NA	476	no
30	12	4*	2;16	0	NA	123	no
31	13	9*	2;>32	4**	2;3	111	yes
32	12	0		1	3	138	no
33	2	1	32	1	32	1320	no
34	7	1*	2	0	NA	187	no

*Indicates the presence of 1 ATU among R isolates.

**Indicates the presence of 2 ATU among R isolates.

^#^ATU differed from the ITC-ATU isolate.

NA, not applicable; ND, not determined; MIC, minimal inhibitory concentration (mg/L).

**Figure 4:**
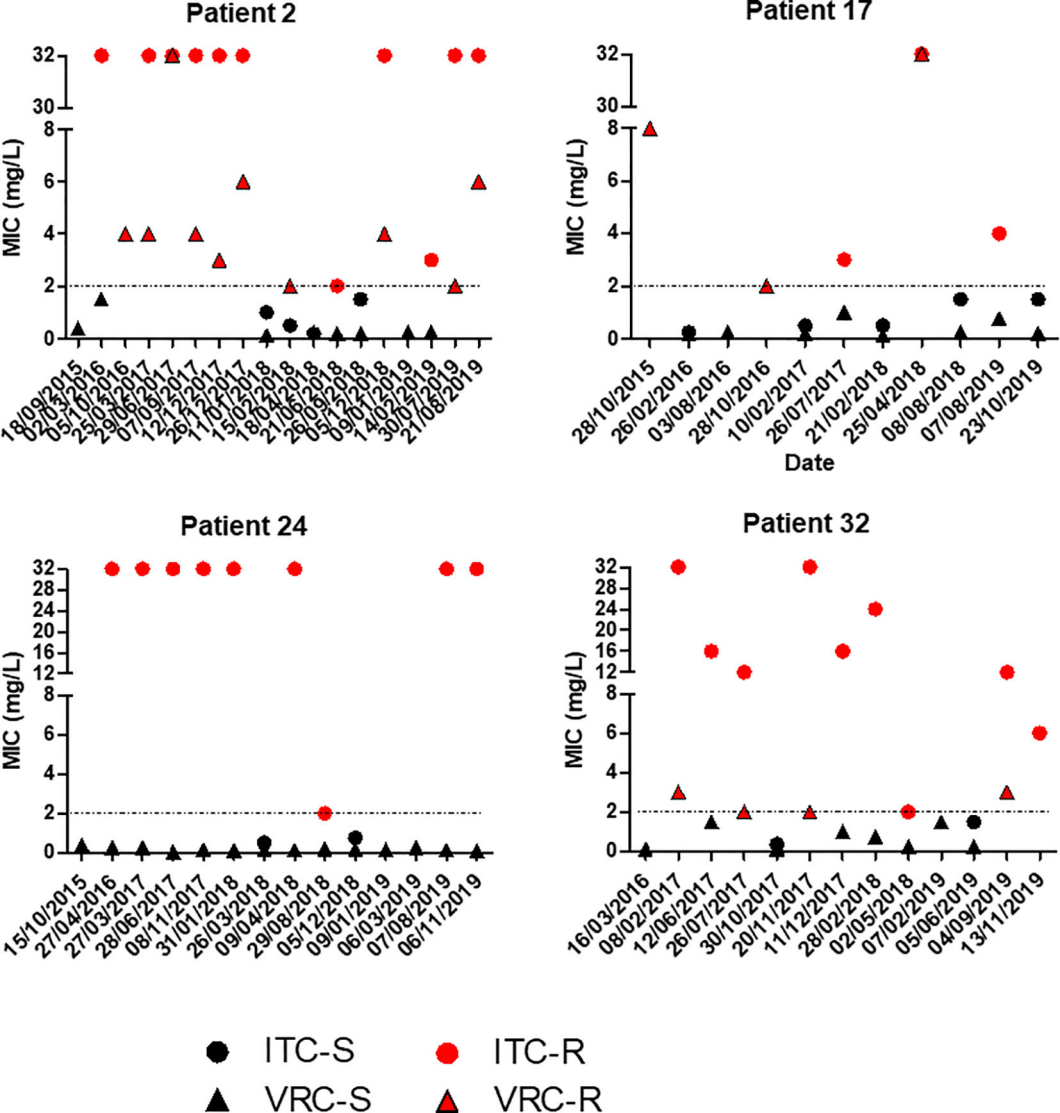
Evolution of itraconazole (ITC) and voriconazole (VRC) MIC values of *A. fumigatus* isolates from four patients in the time. For clarity, isolates with ATU MIC values were gathered with resistant isolates.

As an example of the multiplicity of strains, distribution of MICs of consecutive isolates from four CF patients with >10 isolates were recorded over time. **Figure 4** shows that for a given patient, MIC values fluctuated over time, with cultures of susceptible and resistant phenotypes, underlining the diversity of *A. fumigatus* strains colonizing the airways of CF patients.

*Cyp51A* genotyping of AR*Af* was performed in strains isolated from six CF patients, reflecting the diversity of genetic alterations related to triazole resistance (**Table 4**). Interestingly, *cyp51A* polymorphism were found in 4/6 isolates. While the multi-azole resistant isolate harbored the L98H mutation together with the tandem repeat TR34 (patient 9), the two ITC-R and POS-R AR*Af* displayed the substitutions M220K and G54R respectively (patients 2 and 24). Finally, the combined F46Y, M172V, and E427K amino acid changes were simultaneously found in the ITC-R isolate. Triazole resistance of isolates from patients #3 and #26 was not linked to *cyp51A* polymorphism (wild type genotype) supporting the existence of other biological mechanisms responsible for triazole resistance.

**Table 4:** Profile of azole resistance in CF patients with *cyp51A*-genotyped AR*Af*.

No of patient	ITC MIC	VRC MIC	POS MIC	*cyp51A* mutations
2	>32	1.5	>32	M220K
3	32	16	0.094	none
9	>32	>32	>32	TR34/L98H
24	>32	0.25	>32	G54R
26	32	8	4	none
30	2	0.75	ND	F46Y, M172V, E427K

ND, not determined; MIC, minimal inhibitory concentration (mg/L).

## Discussion

The burden of fungal diseases due to *Aspergillus* remains high in France, with multiple forms, from chronic or allergic to invasive aspergillosis (Gangneux et al., 2016). Furthermore, the emergence *Aspergillus fumigatus* isolates resistant to azoles led international experts to recommend the *in vitro* susceptibility screening of isolates and to shift from azole to amphotericin B as first-line treatment of IA when resistance is higher than 10% (Verweij et al., 2015; Ullmann et al., 2018).

Here we analyzed the results of *in vitro* susceptibility of *A. fumigatus* during a 5-year survey in a routine practice. Because the micro-dilution method recommended by EUCAST is difficult to use in routine when numerous isolates are tested per day, we use Etest^®^ strips and RPMI medium in our routine practice as in many hospitals. The global level of resistance of isolates to voriconazole and itraconazole was 4.1 and 14.5%, respectively. This level remained somewhat stable over years. Interestingly, a recent publication from the Netherlands showed that the VRC-resistance frequency was 34% lower in 2018 than in 2013 (p = 0.0001) (Lestrade et al., 2020). However, our results show that the level of resistance greatly varied according to azole drug, patient origin and clinical setting. Chronic respiratory diseases and particularly cystic fibrosis are favorable to AR*Af* emergence because of recurrent treatment with azoles. Besides, we detected only a very low number of AR*Af* during invasive pulmonary aspergillosis.

Regarding the management of IPA, a few other French papers from Paris area reported similar low levels of ARAf detection (Alanio et al., 2011; Alanio et al., 2016). At the time of hospitalization in Hematology, none of the patients had an ARA*f* isolated, probably linked to the short course of chemoprophylaxis with posaconazole. These results comfort us in the use of azoles as first-line curative drugs (VRC and ISA) or prophylaxis (POS).

Regarding CF patients during 2015–2019, we observed a higher frequency of AR*Af* (11%) than in other French studies. Our center is one of the two tertiary hospitals of the Brittany region which is mainly rural with an intense agricultural and farming activity that could account for high azole resistance rate, and furthermore 16/30 patients with ARA*f* had a pre-exposure to azoles. In the West of France near Brittany, Morio et al., (2012) reported a 8% prevalence of azole resistance in 2010–2011, while this rate was 6.8% four years later, mainly due to a genotypic mutation TR34/L98H (Lavergne et al., 2019). In a cohort from Paris, 4.8% of resistant isolates were detected (Burgel et al., 2012), whereas in a mixed cohort of CF and immunocompromised patients from the North of Paris, only 1.8% of AR*Af* were detected (Choukri et al., 2015). European studies showed that 5.3% (101/2888) of *A. fumigatus* isolates were azole-resistant in a prospective multicenter study in Germany (Seufert et al., 2018). The frequency was 7.1% in the Netherlands, with TR34/L98H being the dominant resistance mechanism (Engel et al., 2019). In a bicentric Italian study, no resistance was detected in one center, while the frequency of resistance was 8.2% in the other (Prigitano et al., 2017). In Denmark, Risum et al. recently reported the occurrence of AR*Af* strains in 7.3% (10/137) CF patients (Risum et al., 2020).

The issue of cross-resistance remains moderate in our center even if azole exposure is frequent, as VRC remains usable for 73% of ITC-R isolates, and a triple-resistance to azole was very scarce. Importantly, isolates that were both ITC-R and VRC-R were POS-R except in one case. Thus, we decided to stop POS *in vitro* susceptibility testing to the benefit of ISA susceptibility testing. Data obtained during this survey incited us to adopt new rules for *in vitro* testing strategy:

(i) When VRC is considered as the first-line treatment: we perform VRC and ISA susceptibility testing and recommend to switch for liposomal amphotericin B when VRC-R and ISA-R. Such situation is possible during invasive, allergic and chronic aspergillosis.
(ii) When ITC is considered as the first-line treatment: we perform ITC, VRC and ISA susceptibility testing and first recommend to change for VRC or ISA in case of ITC-R, and to consider liposomal amphotericin B in case of pan-azole-R. Such situation is possible mainly during allergic and chronic aspergillosis.

Finally, during CF, we confirmed the high diversity of *Aspergillus* isolates. Indeed, CF patients may be colonized with a wide succession of different phenotypes, either susceptible or resistant. This finding could be confirmed by a genotypic characterization of isolates, since it has been described that different genotype, until 5 to 10, could be isolated in a same patients (Neuvéglise et al., 1997; Amorim et al., 2010). Here again, this result made us change our routine procedure to a systematic antifungal susceptibility testing on various colonies from the same samples, as recommended by international guidelines (Ullmann et al., 2018). And as shown by Lestrade et al. (2020), the monitoring of genotypes may also be combined to *in vitro* susceptibility determination since they reported that the mean VRC MIC of TR34/L98H isolates decreased from 8 mg/L (2013) to 2 mg/L (2018) in the Dutch experience.

Overall, this survey underlines a huge variability of azole resistance level depending on the azole drug, the patient origin and the clinical setting, and also variability within a patient. In addition to epidemiological data, this study made us changing our routine procedure of azole *in vitro* testing. The limits of this field and practical study are its monocentric design, some incomplete data for some patients, and the absence of *in vitro* data on isavuconazole that was only recently introduced in our centre.

## Data Availability Statement

The raw data supporting the conclusions of this article will be made available by the authors, without undue reservation.

## Ethics Statement

The studies involving human participants were reviewed and approved by Comité d’éthique de Rennes, France. Written informed consent from the participants’ legal guardian/next of kin was not required to participate in this study in accordance with the national legislation and the institutional requirements.

## Author Contributions

All authors listed have made a substantial, direct and intellectual contribution to the work, and approved it for publication.

## Conflict of Interest

J-PG received research grants from Gilead, MSD, and Pfizer.

The remaining authors declare that the research was conducted in the absence of any commercial or financial relationships that could be construed as a potential conflict of interest.
